# An assessment of COVID-19 vaccine knowledge and acceptability among healthcare workers in a Nigerian tertiary hospital

**DOI:** 10.4314/gmj.v57i4.5

**Published:** 2023-12

**Authors:** Iriagbonse I Osaigbovo, Esohe O Ogboghodo, Otaniyenuwa E Obarisiagbon, Darlington E Obaseki

**Affiliations:** 1 Department of Medical Microbiology, University of Benin Teaching Hospital, PMB 1111, Benin City, Edo State; 2 Department of Public Health and Community Medicine, University of Benin Teaching Hospital, PMB 1111, Benin City, Edo State; 3 Office of the Chief Medical Director/ Department of Anatomic Pathology, University of Benin Teaching Hospital, PMB 1111, Benin City, Edo State

**Keywords:** Healthcare worker, COVID-19, Vaccine, Nigeria, Vaccine hesitancy

## Abstract

**Objectives:**

To assess the knowledge and acceptability of COVID-19 vaccines among HCWs.

**Design:**

A descriptive cross-sectional study was conducted in March 2021 among eligible HCWs using a self-administered questionnaire.

**Setting:**

The study was conducted in a southern Nigerian tertiary hospital.

**Participants:**

All HCWs not on annual or study leave were eligible to participate. The number of HCWs in each occupational category was determined by proportional allocation. HCWs were selected by stratified sampling technique.

**Main outcome measures:**

Knowledge of COVID-19 vaccines was assessed using 25 questions. The minimum and maximum scores were 0 and 25, respectively. Scores were converted to percentages. Scores of 50% and above were rated as good knowledge. Participants were also asked if they were willing to receive the vaccine.

**Results:**

The mean age of 512 participating HCWs was 33.4±7.8 with an M:F ratio of 1:1.1. Overall, 399 (76.6%) had good knowledge. Occupation and exposure to COVID-19 were predictors of knowledge. Three hundred and twenty-eight respondents (63.0%) were willing to take the vaccine. Predictors of willingness to accept vaccination were age, sex, number of years in employment and knowledge about the vaccines (p< 0.05).

**Conclusions:**

Most HCWs had good knowledge and were disposed to accepting the COVID-19 vaccine. Educational interventions are necessary to improve HCWs knowledge as they may provide vaccine-related information to the general public.

**Funding:**

None declared

## Introduction

More than one year after being declared a pandemic, coronavirus disease 2019 (COVID-19) remains a global public health crisis, with more than 100 million people affected worldwide and over 2 million dead.^1^ Efforts to lessen the health and socio-economic impacts of the pandemic have hinged largely on prevention because a definitive antiviral treatment has proven elusive.^2^,^3^ While social distancing, wearing of face masks, hand washing and avoidance of crowded spaces have been the mainstay of containment, compliance rates for these non-pharmacological interventions vary and have not been sufficient to halt the pandemic.^2^, ^4^ Recent estimates show that 60-75% of individuals need to be immune to impede onward transmission and community spread of severe acute respiratory syndrome coronavirus 2 (SARS-CoV-2), the virus that causes COVID-19. 5 The deployment of safe, effective and affordable vaccines with high coverage rates offers the only means of achieving sufficient immunity to lessen morbidity, mortality and finally contain the pandemic.

Rapid and unprecedented efforts have been channelled into developing and testing vaccines against SARS-CoV-2.^6^ At least five vaccines approved for emergency use are currently being rolled out in mass vaccination campaigns with over a billion doses administered worldwide.^7^,^8^

Despite this remarkable progress, the end goal of achieving herd immunity is continuously threatened by individuals and groups choosing to delay or refuse vaccination.^8^ This delay in acceptance or outright refusal of vaccination despite the availability of vaccination services, termed vaccine hesitancy, has been identified by the World Health Organisation (WHO) as one of the ten leading threats to global health. 9,10 Vaccine hesitancy has been described even among healthcare workers (HCWs), including doctors and nurses.^11^ In India, for instance, a significant proportion of HCWs and other eligible ‘frontliners’ have not turned up for their second dose of the COVID-19 vaccine.^12^

HCWs are at high risk of contracting SARS-CoV-2 from inapparent, suspected or confirmed COVID-19 patients.^13^ Thus, the World Health Organisation has listed them as a priority group for COVID-19 vaccination.^14^ Vaccinating this vulnerable group protects the HCWs themselves, their household contacts, their patients and invariably, the healthcare systems where they operate.^11^ Unfortunately, COVID-19 vaccine hesitancy amongst HCWs has been widely documented, ranging from 4-72% and averaging 22.1% in a systematic review conducted in February 2021.^15^ If vaccination efforts are to succeed, vaccine hesitancy among HCWs must be continually assessed and addressed by governments, public health authorities and even health facilities at the grassroots. Gauging knowledge and acceptability among HCWs will determine uptake and the likelihood that they will recommend the vaccines to patients. To enable the management of a tertiary healthcare facility determine intervention measures necessary for successful vaccine roll-out and coverage, a survey was conducted to assess HCWs' knowledge about available COVID-19 vaccines and willingness to accept them.

## Methods

### Study design

This descriptive cross-sectional study was part of a larger study assessing knowledge, attitudes and willingness to accept COVID-19 vaccination among HCWs in the University of Benin Teaching Hospital (UBTH), Benin City, Edo State, Nigeria.

### Setting

The UBTH is an 850-bed tertiary hospital with a staff strength of 4220, providing promotive, preventive, curative and rehabilitative services in various sub-specialties.^16^ It is also a designated isolation and treatment facility for COVID-19 and provides diagnostic services for COVID-19 in the state by its molecular virology laboratory.^17^,^18^ Since the onset of the pandemic, the management of the facility has prioritised staff welfare and safety by risk-stratifying staff and exempting those at high risk of severe COVID-19 from patient-facing tasks; active surveillance for COVID-19 infection in healthcare personnel within the facility and providing personal protective equipment sourced both conventionally and through indigenous production.^17^,^19^,^20^ Ensuring that staff are vaccinated against the virus is a natural extension of this managerial responsibility. The study was conducted in March 2021, just before vaccine roll-out commenced in the state.

### Study population

The study population included all cadres of HCWs, excluding those on annual or study leave. An HCW was defined as any member of staff in the health care facility involved in the provision of patient care, including those who have been present in the same area as patients as well as those who may not have provided direct care to patients but who have had contact with patient's body fluids, potentially contaminated items or environmental surfaces. These included doctors, nurses, pharmacists, medical laboratory scientists, and administrative staff. 21

### Sample size determination and sampling method

A minimum sample size of 394 was calculated using the appropriate formulae for single proportion.^22^ This was calculated considering a standard normal deviate of 1.96 at a significance level of 5%; p of 36.0% (representing the prevalence of COVID-19 acceptability among HCWs in the United States)^23^ and a 10% attrition rate (non-response).

HCWs were selected using a stratified sampling technique. Employee cadre formed the basis of each stratum. Proportional allocation was used to determine the number of employees in each occupation. A systematic sampling technique was used to select the respondents from each occupation. A sampling interval was calculated using the list of registered employees in each occupational group as a sampling frame. The first respondent was selected using a simple random sampling method, after which every n^th^ respondent was selected till the required sample size was achieved.

### Data collection

Twelve research assistants were recruited and underwent a one-day training on the purpose of the study, their roles during the study period and the sampling technique to be used. The research assistants were needed to facilitate the completion of data collection in the short interval between the commencement of the study and the vaccine roll-out in the facility. Clear explanations of the various sections in the semi-structured questionnaire employed were given.

Data were collected using a self-administered questionnaire derived from a presentation by the Centres for Disease Control, Atlanta, United States of America COVID-19 Response Vaccine Task Force, titled ‘COVID-19 Vaccine Basics: What Healthcare Personnel Need to Know’.^24^ The questionnaire required socio-demographic characteristics of the respondent, awareness about COVID-19 vaccines, respondent's experience with COVID-19. It also assessed factual knowledge about COVID-19 vaccines, willingness to accept the vaccine, and the reasons for non-acceptance in those unwilling.^24^ A detailed explanation was given to all eligible respondents on the purpose of the study, and informed consent was sought before the administration of the questionnaire.

### Data analysis and management

The filled questionnaires were checked for completeness and consistency by the researcher and given identification codes before being entered into IBM SPSS version 25.0 for analysis. Knowledge of COVID-19 vaccine was assessed using 25 questions in 12 domains. A correct response was scored 1, and an incorrect answer scored 0. Scores were converted to percentages, and modified Bloom's cut-off points were used to judge knowledge: scores 50% and above were adjudged as good, while scores less than <50% were adjudged poor.^25^ The questions for scoring Knowledge of COVID-19 vaccine were assessed for internal consistency and reliability using the Cronbach's alpha test, with a value of 0.783. Acceptability of the COVID-19 vaccine was assessed using respondents' replies on whether or not they were willing to be vaccinated.

Unadjusted and adjusted analyses using binary logistic regression were conducted using the ‘enter approach’ to determine significant predictors of knowledge and willingness to accept the COVID-19 vaccine. The statistical measure for the analysis was the adjusted odds ratio and 95% confidence interval. The significance level was set at p < 0.05 for all statistical associations. Frequency tables and figures were used to present the results.

### Ethical considerations

Ethical approval for this study was obtained from the Ethics and Research Committee of the University of Benin Teaching Hospital (Protocol number ADM/E22/A/VOL.VII/14831026). Respondents were informed of their rights to decline participation or withdraw from the study whenever they wished. Informed consent, privacy and confidentiality were assured.

## Results

Five hundred and seventy questionnaires were distributed. Of these, 521 were filled by respondents, giving a response rate of 91.4%. Survey respondents were 271 (52.0%) females and 250 (48.0%) males, giving a male: female ratio of 1:1.1. A higher proportion of respondents, 212 (40.7%), were in the 30–39-year age bracket and had spent ≤ 5 years in employment 360 (69.1%), while most, 509 (97.7%) were Christians and had tertiary education 516 (99.0%) (Table 1)

**Table 1:** Socio-demographic characteristics of respondents

Variables	Frequency (n%)
**Age group (years)**	
**20-29**	192 (36.9)
**30-39**	212 (40.7)
**40-49**	95 (18.2)
**50-59**	22 (4.2)
**Mean ± SD**	**33.4 ±7.8**
**Sex**	
**Female**	271 (52.0)
**Male**	250 (48.0)
**Religion**	
**Christian**	509 (97.7)
**Muslim**	12 (2.3)
**Marital status**	
**Married**	268 (51.4)
**Single**	253 (48.6)
**Highest level of education**	
**Secondary**	5 (1.0)
**Tertiary**	516 (99.0)
**Cadre**	
**Doctor**	244 (46.8)
**Nurse**	119 (22.8)
**Administrative Staff**	60 (11.5)
**Pharmacist**	56 (10.7)
**Medical Lab Scientist**	27 (5.2)
**Others** *	15 (2.9)
**Years of employment**	
**≤ 5**	360 (69.1)
**6 - 10**	83 (15.9)
**11 - 15**	45 (8.6)
**16 - 20**	20 (3.8)
**21 - 25**	9 (1.7)
**> 25**	4 (0.8)
**Mean ± SD**	**5.0 ± 5.4**

* Others include Paramedics, Health Assistants and Physiotherapists

Almost all the respondents, 517 (99.8%), had heard about COVID-19 vaccines. Respondents got information about the vaccines from multiple sources, mainly from the internet 389 (74.7%) and television 361 (69.3%). Two hundred and forty-five (47.0%) obtained information about the vaccines at the workplace. Other sources of information were radio 186 (35.7%), newspaper 177 (34%), friends 167 (32.1%), and religious houses 64 (12.3%).

The respondents' experience with COVID-19 is shown in Table 2. The majority, 316 (60.7%), had been exposed to COVID-19 patients, while 107 (20.0%) had been infected. Of those infected (n=107), 81 (75.7%) received home-based care, while 26 (24.3%) were hospitalised. Most respondents, 400 (76.8%), knew someone infected, and 266 (67.0%) reported that this was a co-worker.

**Table 2:** The COVID-19 experience of respondents

Variable	Frequency (n%)
**Exposure to a COVID-19 patient (n = 521)**	
**Yes**	316 (60.7)
**No**	205 (39.3)
**Awareness of someone infected with COVID-19 (n = 521)**	
**Yes**	400 (76.8)
**No**	121 (23.2)
**Person infected** * **(n =400)**	
**Co-worker**	266 (66.5)
**Friends**	145 (36.3)
**Other relatives**	49 (12.3)
**Neighbour**	27 (6.8)
**Sibling**	22 (5.5)
**Spouse**	9 (2.3)
**Child**	1 (0.3)
**Ever infected with Covid-19 (n = 521)**	
**Yes**	107 (20.5)
**No**	414 (79.5)
**Mode of Treatment (n = 107)**	
**Home-based care**	81 (75.7)
**Hospital**	26 (24.3)

* Multiple responses

### Knowledge of COVID-19 vaccines

The responses to various domains of knowledge about COVID-19 vaccines is shown in Table 3. Only 29 (5.6%) of respondents knew that COVID-19 vaccines do not interact with our DNA in any way, and 50 (9.6%) and 54 (10.4%) knew the types of vaccines that were available and the amount of time needed for immunity to develop, following receipt of the vaccine respectively. Overall, 399 (76.6%) had good knowledge, while 122 (23.4%) had poor knowledge. The mean (standard deviation) knowledge score was 14.57 ± 2.86

**Table 3:** Knowledge of COVID-19 vaccine (N=521)

Knowledge Variables	Correct response (n%)
**Knowledge of characteristics COVID-19 Vaccine**	
**The vaccine could be an mRNA vaccine that protects against SARS-CoV-2**	385 (73.9)
**Vaccine is a cure for Covid-19**	432 (83.1)
**COVID-19 Vaccines contain the live virus**	489 (93.9)
**COVID-19 Vaccines do not interact with our DNA in anyway**	29 (5.6)
**COVID-19 vaccines can give someone COVID-19 Knowledge of the importance of COVID-19 vaccination**	501 (96.4)
**Vaccination will help protect from COVID-19**	356 (68.9)
**COVID-19 vaccination is one way to end the pandemic**	184 (35.3)
**Vaccination will not create immune response against the virus**	452 (86.8)
**Additional protective measures are not necessary after vaccination**	508 (97.5)
**Types of vaccines available**	50 (9.6)
**Category of priority persons to get the COVID-19 vaccine**	
**Frontline Health workers**	376 (72.2)
**Elderly**	217 (41.7)
**Children**	100 (19.2)
**The COVID-19 vaccine can be taken even if one has already been infected by the virus**	327 (62.8)
**The COVID-19 vaccine is safe like all vaccines**	213 (40.9)
**Duration of protection following COVID-19 vaccination**	183 (35.1)
**Immunity following vaccination occurs after a few months**	54 (10.4)
**Side effects following vaccination are signs that the body is building immunity against the virus**	211 (40.5)
**Vaccine adverse event reporting system, V-safe and others are used to monitor vaccine safety**	308 (59.1)
**Common AEFI of COVID-19 vaccination include**	
**Fever**	353 (67.8)
**Muscle aches**	239 (45.9)
**Headaches**	205 (39.3)
**Vomiting**	157 (30.1)
**Obesity**	515 (98.8)
**Overall Knowledge score**	
**Good**	399 (76.6)
**Poor**	122 (23.4)
**Mean (SD) Knowledge Score**	14.57 ± 2.86

AEFI = Adverse Events Following Immunisation

Table 4 shows the unadjusted and adjusted association between socio-demographic variables, exposure to and infection with COVID-19 and knowledge of COVID-19 vaccines. Upon adjustment, the sex, occupation of HCW and exposure to a COVID-19 patient were the significant independent predictors of good knowledge of COVID-19 vaccines. Females, HCWs besides medical doctors and HCWs who were exposed to a COVID-19 patient were 1.7 times less likely to have good knowledge compared to others. Although respondents who were 35 years or younger and had worked 5 years or less in the facility were also more likely to have good knowledge of COVID-19 vaccines, these were not statistically significant.

**Table 4:** Unadjusted and Adjusted predictors of knowledge of COVID-19 vaccination

Predictors	Unadjusted OR (95% CI)	p-value	Adjusted OR (95% CI)	p-value
**Age Group (years)**				
**≤ 35**	1.253 (0.829 - 1.895)	0.284	0.710 (0.411 - 1.228)	0.221
**> 35** *	1		1	
**Sex**				
**Male**	1.792 (1.211 - 2.654)	0.003	0.633 (0.417 - 0.963)	0.032
**Female** *	1		1	
**Religion**				
**Christian**	1.105 (0.295 - 4.141)	0.882	0.933 (0.238 - 3.655)	0.921
**Muslim** *	1		1	
				
**Marital Status**				
**Married**	0.961 (0.652 - 1.416)	0.841	0.832 (0498 - 1.390)	0.482
**Single** *	1		1	
**Cadre**				
**Medical Doctor**	2.181 (1.468 - 3.240)	< 0.001	0.578 (0.371 - 0.899)	0.015
**Other HCWs** *	1		1	
**Years of Employment**				
**≤ 5**	1.280 (0.832 - 1.967)	0.260	1.016 (0.600 - 1.720)	0.952
**> 5** *	1		1	
**Exposure to Covid-19 Patients**				
**Yes**	1.910 (1.256 - 2.903)	0.002	0.615 (0.394 - 0.962)	0.033
**No** *	1		1	
**Ever infected with Covid-19**				
**Yes**	1.507 (0.949 - 2.395)	0.081	0.755 (0.465 - 1.228)	0.257
**No** *	1		1	

* Reference category

### Willingness to accept COVID-19 vaccines

About two-thirds of the HCWs 328 (63.0%) were willing to take the COVID-19 vaccine when available while 193 (37.0%) were not. Among those unwilling to take the vaccines, the reasons proffered were that side effects of the vaccine are not fully understood in 124 (64.3%); efficacy of the vaccine is not yet known in 97 (50.3%) and the vaccine was made too quickly in 71 (36.8%), amongst others. (Table 5).

**Table 5:** Willingness to take the vaccine

Variable	Frequency (n%)
**Will Take the Vaccine if Available**	
**Yes**	328 (63.0)
**No**	193 (37.0)
If No, reasons* **(n=193)**	
**Side effects of the vaccine are not fully understood**	124 (64.3)
**Efficacy of the vaccine is not yet known**	97 (50.3)
**The vaccine was made too quickly with not enough time to understand the effectiveness**	71 (36.8)
**The vaccine does not provide immunity against new strains or variants of the virus**	48 (24.9)
**Religious**	11 (5.7)

* Multiple responses

Table 6 shows the unadjusted and adjusted association between socio-demographic variables, exposure to and infection with COVID-19 and willingness to take COVID-19 vaccines. Upon adjustment, age [AOR 2.239 (95% CI- 1.176 - 4.265)], sex [AOR 0.572 (95% CI- 0.357 - 0.918)], years of service of the respondents [AOR 0.526 (95% CI- 0.290 - 0.954)], and knowledge of COVID-19 vaccines [AOR 0.345 (95% CI- 0.194 - 0.613)] had statistically significant association with willingness to take COVID-19 vaccine (p < 0.05).

**Table 6:** Unadjusted and Adjusted predictors of willingness to take the COVID-19 vaccination

Predictors	Unadjusted OR (95% CI)	p-value	Adjusted OR (95% CI)	p-value
**Age Group (Years)**				
**≤ 35**	1.206 (0.832 - 1.746)	0.322	2.239 (1.176 - 4.265)	0.014
**> 35** *	1		1	
**Sex**				
**Male**	2.291 (1.588 - 3.307)	< 0.001	0.572 (0.357 - 0.918)	0.021
**Female** *	1		1	
**Religion**				
**Christian**	0.560 (0.150 - 2.093)	0.382	1.434 (0.304 - 6.773)	0.649
**Muslim** *	1		1	
**Marital Status**				
**Married**	0.775 (0.542 - 1.107)	0.161	1.149 (0.654 - 2.018)	0.629
**Single** *	1		1	
**Cadre**				
**Medical Doctor**	2.256 (1.561 - 3.260)	< 0.001	0.768 (0.465 - 1.268)	0.303
**Other HCWs** *	1		1	
**Years of Employment**				
**≤ 5**	1.857 (1.270 - 2.715)	0.001	0.526 (0.290 - 0.954)	0.034
**> 5** *	1		1	
**Exposure to Covid-19 Patients**				
**Yes**	0.968 (0.672 - 1.394)	0.861	1.161 (0.714 - 1.890)	0.547
**No** *	1		1	
**Infected with Covid-19**				
**Yes**	1.028 (0.658 - 1.605)	0.905	1.199 (0.681 - 2.112)	0.529
**No** *	1		1	
**Knowledge of Covid-19 vaccines**				
**Good knowledge**	4.368 (2.655 - 7.186)	< 0.001	0.345 (0.194 - 0.613)	< 0.001
**Poor knowledge** *	1		1	

* Reference category

## Discussion

In the ongoing COVID-19 vaccination campaign, initial uptake by HCWs is critical for safety, health system functioning, and public opinion.^25^ Thus, HCWs constitute a priority group capable of influencing the trajectory of vaccine roll-out. Weeks before the Oxford-Astra Zeneca vaccine roll-out in Edo state, southern Nigeria, this study showed that about three-quarters of HCW respondents had good knowledge about COVID-19 vaccines. Despite the good number with sufficient knowledge, only two-thirds were willing to receive the vaccine.

Compared with the general public, HCWs are expected to have evidence-based information on vaccines, such as the mode of action, logistics of vaccination and side effect profile.^26^ It is, thus, not surprising that the majority had good overall knowledge about COVID-19 vaccines. Nevertheless, this survey revealed gaps in respondents' factual knowledge, which health education needs to address. For instance, only about 6% were sure that ‘COVID-19 vaccine does not interact with our DNA in any way’. HCWs should be knowledgeable enough to dispel such myths, which can fuel the rejection of vaccines among members of the general public. Knowledge of the common adverse events following immunisation (AEFI) expected with COVID-19 vaccines, such as muscle aches, was also poor. It is difficult to say if the general knowledge observed here is peculiar to the study setting because studies assessing factual knowledge about COVID-19 vaccines are rare. We observed that sex, occupation and exposure to COVID-19 were the strong predictors of knowledge about COVID-19 vaccines, with males, doctors and exposed HCWs having better knowledge. More studies calibrating the knowledge of HCWS are needed in other locales as lay individuals depend on them to dispel myths and counter misinformation.

A lower proportion of HCWs were willing to be vaccinated than those with good knowledge. However, this exceeded 40-58% willing to be vaccinated in the general Nigerian population.^27^-^29^ This is not surprising as HCWs, expectedly, are more knowledgeable about vaccines and vaccine-preventable diseases than the general public; indeed, good knowledge was a predictor of willingness to be vaccinated in this study.

In some local population studies 30,31, willingness to receive a prospective vaccine was also associated with being a HCW.

However, a national online survey found no significant difference between HCWs and the general public.^29^ The time the studies were conducted may also have influenced the acceptability of the vaccine. While the general population studies mentioned were conducted in 2020 when vaccines were still undergoing clinical trials or had not been authorised for emergency use, the index survey was conducted in 2021 when 20 million doses had already been administered in the United Kingdom alone.^32^ Thus, witnessing ongoing vaccination worldwide may have positively impacted the willingness to be vaccinated in the index study's participants. A higher sense of risk perception may also have influenced HCWs willingness to be vaccinated compared to the general public.

Only a few researchers have explored Nigerian HCWs' willingness to receive the COVID-19 vaccine.^33^ The number willing to be vaccinated in this study is higher than 53.5% observed in the online survey conducted by Ekwebene *et al.* 33, but this may be due to the wider coverage of the latter survey, which included HCWs in all geo-political zones and cadres of health facility in Nigeria.^33^ The period in which the survey was conducted was not indicated, so the effect this may have had on the results is not apparent.

Compared to other African countries, this survey's self-reported willingness to get vaccinated far surpassed the 21% reported in Egyptian HCWs.^34^ Likewise, it exceeded observations of 28% and 39% reported in HCWs from Democratic Republic of Congo (DRC) and Ghana, respectively.^35^,^36^ Compared to Nigeria, these sub-Saharan countries have a lower burden of COVID-19 and this may have affected HCWs willingness to be vaccinated. Also, the DRC survey was conducted in March to April, 2020 when the full impact of the pandemic may not have been obvious to respondents, and there was as yet no vaccine undergoing clinical trials.^35^ Although not a significant predictor in our study, the COVID-19 experiences of HCWs in the various locales may also have played a determining role as other studies have shown greater acceptance rates of the vaccine among HCWs who had been in contact with or cared for COVID-19 patients.^37^,^38^

In the Ghanaian study, a significantly higher proportion of the HCWs who had not been in contact with COVID-19 patients were unwilling to accept the vaccines than those who had.^36^

Given that 60.7% of the Ghanaian HCWS reported no contact with COVID-19 patients^36^, the converse of what was obtained in their Nigerian counterparts, it is unsurprising that willingness to accept the vaccine was higher in the latter group.

Generally, HCWs outside Africa were more willing to accept the COVID-19 vaccine than in the index study-74% in France, 70% in Saudi Arabia and 76% in Israel.^11^,^37^,^38^ However, acceptability ranged from 36%-58% in the United States (US), lower than observed in our survey.^23^,^39^ The politicisation of the US governmental pandemic response in 2020, which ignited public mistrust of authorities, may have resulted in fewer HCWs willing to be vaccinated.^25^

The finding in this study that sex predicted acceptability with male HCWs more willing to be vaccinated is in tandem with observations from similar studies conducted in other locations.^35^,^36^,^38^,^39^ The globally established fact that males have a higher risk of severe disease and mortality compared to females may be responsible for this.^40^ Another possible explanation may be the widespread myth about COVID-19 vaccines affecting female fertility. General population studies also found that females are less likely to accept a COVID-19 vaccine, possibly due to concerns about side effects such as infertility, serious side effects making them unable to take care of families, or greater susceptibility to myths and misinformation from media.^15^ Similar perceptions, although not assessed for in this survey, may have influenced the decision of female HCWs. Furthermore, there is a lack of safety and efficacy data in pregnancy as pregnant women were excluded from phase III clinical trials of the vaccines.^41^ This may have further discouraged acceptability amongst some female HCWs, as respondents were mostly in the reproductive age group. The other predictors of acceptability in this study- age and occupational category- have also been corroborated by other researchers, with older HCWs and medical doctors being more willing to be vaccinated.^15^

To boost the uptake of vaccines, it is important to address the concerns of more than a third of HCWs who expressed unwillingness to be vaccinated. This proportion was higher than the global average of about 23% computed in a scoping review of studies on vaccine hesitancy in HCWs between February 2020 and February 2021.^15^ As in similar surveys, worries about side effects and safety were the most popular reasons for unwillingness to receive the vaccine. Other concerns were efficacy and whether the vaccines would be effective against emerging variants of SARS-CoV-2. Similar to the findings of a survey conducted in the United States, religious beliefs were rarely a concern among HCWs unwilling to be vaccinated.^39^ This may be attributed to the predominantly Christian demographic found in this survey, as refusal of other vaccines has been reported more extensively in Muslim populations in Nigeria.^42^

Unlike previous surveys, which queried the acceptability of a prospective vaccine, this survey is unique because it assessed respondents' knowledge and willingness to take COVID-19 vaccines in real-time when actual vaccination roll-out had commenced in other parts of the world and was imminent in Nigeria. In addition, it is one of only a few which have assessed factual knowledge about COVID-19 vaccines; most other KAP studies only assessed whether participants were aware of the vaccine or knowledge of COVID-19. Probability sampling techniques are another strength that gives this survey the advantage of being more representative of the targeted population compared to the more predominantly employed non-probabilistic methods, which are more prone to self-selection bias.

The survey also has some limitations. The cross-sectional design precludes the establishment of causality since it was conducted at a single point in time. In contrast, the pandemic is dynamic, with information, options and perceptions changing rapidly. For instance, after the survey, reports surfaced of a rare but potentially fatal vaccine-induced immune thrombotic thrombocytopenia associated with Oxford-AstraZeneca COVID-19 vaccine.^43^ Without adequate contextualisation and clarification, such information may negatively affect willingness of HCWs to receive the vaccine in future. Secondly, the survey was targeted at a specific population in a single healthcare institution in the south-south geo-political zone and so the results may not be applicable to other parts of Nigeria.

Besides, the effect of some demographic characteristics such as educational level and religion could not be explored because the sample population was almost uniform in these characteristics.

## Conclusion

This survey revealed good knowledge about COVID-19 vaccines among HCWs working at a tertiary hospital in southern Nigeria providing isolation and treatment for COVID-19 patients. The majority of the HCWs were also willing to be vaccinated against the disease in a proportion exceeding what has been documented in other African countries. Surveillance for adverse events following vaccination and dissemination of accrued local data may help to reassure unwilling staff about vaccine safety, thereby improving uptake.
